# Use of diagnosis codes for detection of clinically significant opioid poisoning in the emergency department: A retrospective analysis of a surveillance case definition

**DOI:** 10.1186/s12873-016-0075-4

**Published:** 2016-02-08

**Authors:** Joseph M. Reardon, Katherine J. Harmon, Genevieve C. Schult, Catherine A. Staton, Anna E. Waller

**Affiliations:** Division of Emergency Medicine, Duke University, 2301 Erwin Rd, Box 3935, Durham, NC 27710 USA; Carolina Center for Health Informatics and the Injury Prevention Research Center, University of North Carolina at Chapel Hill, 100 Market St, Chapel Hill, 27516 NC USA; Department of Emergency Medicine, University of North Carolina at Chapel Hill, Box 7594, 170 Manning Dr, Chapel Hill, 27599 NC USA; Duke Global Health Institute, Duke University, 310 Trent Dr, Durham, 27710 NC USA

**Keywords:** Opioid, Narcotic, Poisoning, Overdose, Emergency department, Surveillance, Diagnosis codes, Naloxone

## Abstract

**Background:**

Although fatal opioid poisonings tripled from 1999 to 2008, data describing nonfatal poisonings are rare. Public health authorities are in need of tools to track opioid poisonings in near real time.

**Methods:**

We determined the utility of ICD-9-CM diagnosis codes for identifying clinically significant opioid poisonings in a state-wide emergency department (ED) surveillance system. We sampled visits from four hospitals from July 2009 to June 2012 with diagnosis codes of 965.00, 965.01, 965.02 and 965.09 (poisoning by opiates and related narcotics) and/or an external cause of injury code of E850.0-E850.2 (accidental poisoning by opiates and related narcotics), and developed a novel case definition to determine in which cases opioid poisoning prompted the ED visit. We calculated the percentage of visits coded for opioid poisoning that were clinically significant and compared it to the percentage of visits coded for poisoning by non-opioid agents in which there was actually poisoning by an opioid agent. We created a multivariate regression model to determine if other collected triage data can improve the positive predictive value of diagnosis codes alone for detecting clinically significant opioid poisoning.

**Results:**

70.1 % of visits (Standard Error 2.4 %) coded for opioid poisoning were primarily prompted by opioid poisoning. The remainder of visits represented opioid exposure in the setting of other primary diseases. Among non-opioid poisoning codes reviewed, up to 36 % were reclassified as an opioid poisoning. In multivariate analysis, only naloxone use improved the positive predictive value of ICD-9-CM codes for identifying clinically significant opioid poisoning, but was associated with a high false negative rate.

**Conclusions:**

This surveillance mechanism identifies many clinically significant opioid overdoses with a high positive predictive value. With further validation, it may help target control measures such as prescriber education and pharmacy monitoring.

## Background

Opioid poisonings have risen precipitously from the 1980s through the 2000s, especially in the south-eastern United States, overtaking motor vehicle collisions as the leading cause of unintentional injury death [1–4]. In 2010, there were 25,036 opioid poisoning-related emergency department (ED) visits in North Carolina (NC), using *International Classification of Disease, 9*^*th*^*edition Clinical Modification* (ICD-9-CM) diagnosis and external cause codes [2]. These figures were almost twofold higher than extrapolations of estimated poisonings from the United States National Electronic Injury Surveillance System (NEISS) [5]. While NEISS is useful for state-level estimates and comparisons of injury and poisoning rates, it does not have the level of detail that is helpful for state public health workers for planning prevention activities. For instance, in 2010, ED visit rates for poisonings had almost tenfold variation between counties, ranging from 4 to 39 ED visits per 10,000 person-years [2]. There is an urgent need for validated tools to track the incidence of nonfatal poisonings.

The NC Disease Event Tracking and Epidemiologic Collection Tool (NC DETECT) collects ICD-9-CM codes and triage data from visits to all 24/7 civilian acute care EDs [6]. The ability of NC DETECT to distinguish the primary cause of an ED visit is often limited because ED visits contain multiple diagnosis codes and it is difficult to interpret the severity or clinical significance of the conditions referenced those codes alone. NEISS, in contrast, collects data directly from a national representative sample of US EDs. Data are entered directly by a trained NEISS representative at each hospital for all reportable injuries in the system, which are determined by a Division of the United States Consumer Product Safety Commission [7] rather than being derived from ICD-9-CM codes. Similar data are collected from a sample of EDs in the United States Drug Abuse Warning Network (DAWN) [8]. This may explain the higher estimates for opioid poisoning in NC DETECT relative to estimates from NEISS or DAWN.

The definition of acute opioid poisoning for disease surveillance has not been clearly established. In public health surveillance, the CDC Guidelines Working Group states that a surveillance system’s utility is determined in part by the degree to which it provides “an improved understanding of public health implications” of adverse health-related events [9]. Most current definitions are not aimed at this type of utility. ICD-9-CM codes 965.** rely on medical coders’ transcription of the diagnosis based on primary clinician documentation [6]. In contrast, DAWN identifies cases using an algorithmic approach but divides cases into overmedication (in which the clinician documented that the patient exceeded a prescribed or recommended dose), adverse reaction (in which the clinician attributed symptoms to a drug side effect), or other toxicity, which do not necessarily correspond to ICD-9-CM coding [10]. The aim of the current initiative was to create a case definition of clinically significant opioid poisoning for surveillance purposes (i.e., those cases in which opioid poisoning prompted an ED visit) and to assess the degree to which ICD-9-CM codes for opioid poisoning correctly identify these opioid poisonings.

## Methods

### Ethics and data use statement

This work was considered exempt from IRB review by the University of North Carolina IRB (12–0448) and by the Duke University IRB (Pro00047085) as a quality improvement initiative. The data used were abstracted from patient charts and thus were not publicly available. Data access was granted by the IRBs and research units of the respective institutions.

### Case definition

ED visits containing one or more of the ICD-9-CM diagnosis codes listed in Table 1 were characterized as either opioid poisoning-related or non-opioid poisoning-related based on the diagnosis code. Non-opioid poisoning diagnosis codes were included as a comparison group.

**Table 1:** ICD-9-CM diagnosis codes included in ED visit sample

Opioid poisoning-related diagnosis codes	Non-opioid poisoning-related diagnosis codes
Poisoning by opium, unspecified (965.00)	Late effect of poisoning due to drug, medicinal or biological substances (909.0)
Poisoning by heroin (965.01)	Poisoning by other specified drugs and medicinal substances (977.9)
Poisoning by methadone (965.02)	Drug dependence (304)
Poisoning by other opiates or related narcotics (965.09)	Opioid abuse (305.5)
Accidental poisoning by heroin (E850.0)	
Accidental poisoning by methadone (E850.1)	
Accidental poisoning by other opiates or related narcotics (E850.2)	

**Fig. 1:**
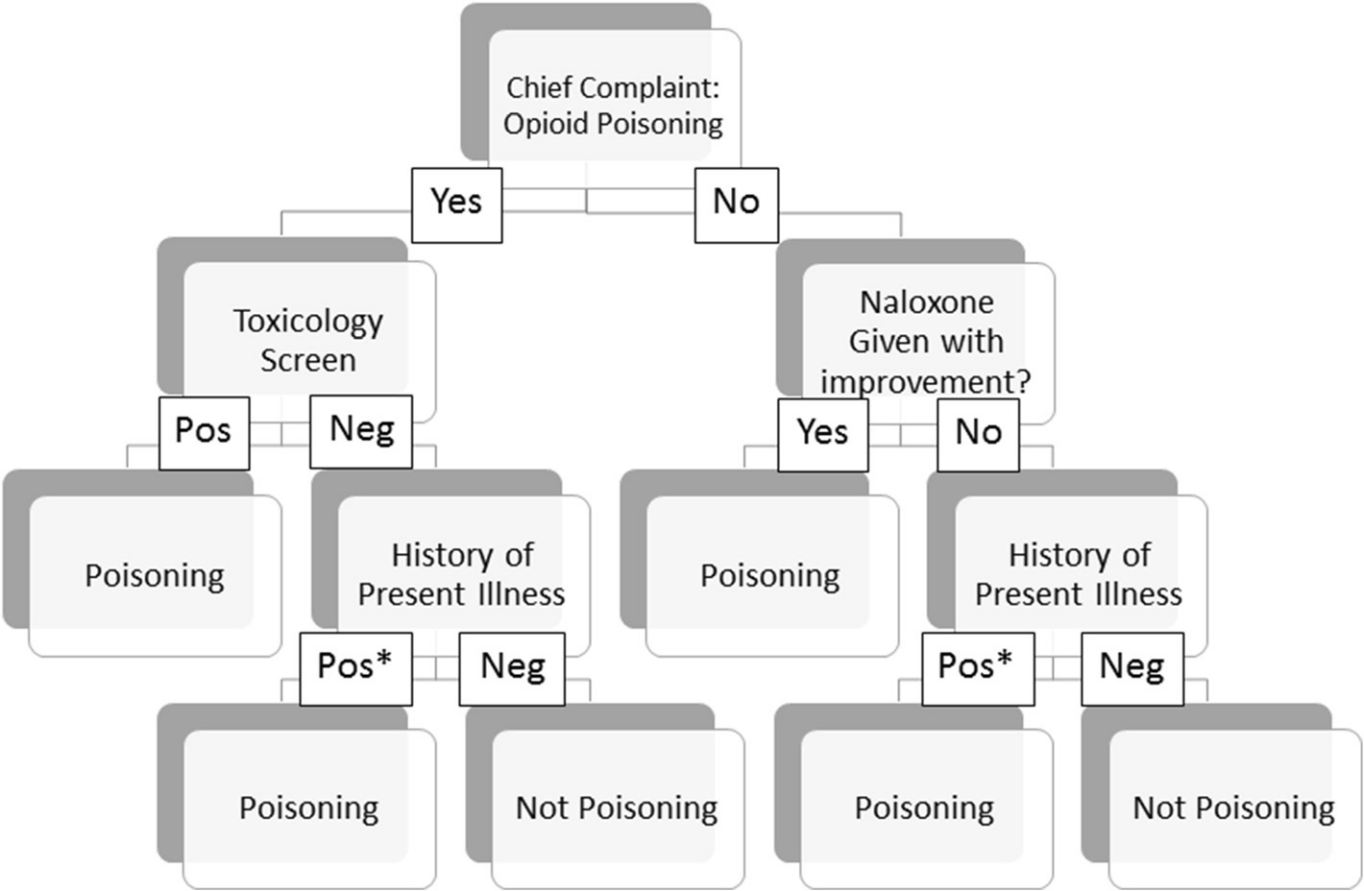
9pt?>Decision tree for identifying clinically significant opioid poisoning in emergency department visits coded as opioid poisoning. *History of Present Illness was positive for opioid poisoning if it included signs of opioid poisoning (altered mental status or respiratory depression) after an opioid ingestion

Using collaborative input from epidemiologists and emergency physicians, the authors developed a case definition *a priori* for clinically significant opioid poisoning and a record review algorithm to efficiently assess whether a case could be considered clinically significant. We defined a visit as a “clinically significant opioid poisoning” if in the judgment of the reviewers, the ED visit would have been averted had the patient not consumed the opioid. In cases of unclear causality (i.e., multiple drugs taken), visits were coded as opioid poisoning unless there was documentation that the primary clinician felt the dose of opioid consumed was too low to have generated the patient’s symptoms. Cases in which the medical decision-making indicated either the clinician’s doubt about whether the patient’s symptoms were due to opioid poisoning, or the clinician’s impression that another agent was primarily responsible for the patient’s symptoms, were not classified as clinically significant poisonings. An algorithm standardized the interpretation of classification of opioid poisoning events (Fig. 1). Because a few patients in the sample had an extended serum opioid toxicology panel sent for analysis, any toxicology test positive for opioids was considered positive for the purposes of the algorithm.

### Data collection

Institutional ED discharge records were used to identify all patients presenting between July 1 2009 and June 30, 2012 at two tertiary care University EDs (University Hospital 1 and 2) and two secondary care Community EDs (both affiliated with University Hospital 2) with opioid poisoning-related diagnosis codes (Table 1). General codes such as 965 (without a decimal modifier) were not queried due to lack of specificity. Due to the large number of cases at University Hospital 2, the authors conducted a simple random sample using JMP 11 (SAS Institute, Cary, NC) to select cases for review. We did not perform independent power calculations for each hospital as three of the hospitals included the entire sample population at their hospital. During the dates sampled, no hospital was undergoing any changes in electronic medical record systems, coding or billing processes. Each hospital sampled uses a staff of professional coders and computer-assisted coding. Data were abstracted independently by two resident physicians (JMR and GCS) who each reviewed the data abstraction method prior to the study. Clinicians reviewed the free text ED visit note, lab tests ordered, psychiatric consult note, and inpatient discharge summary (if applicable) for each visit. Data evaluated included reported history of drug poisoning, patient disposition (hospital admission or discharge), clinical symptoms, toxicology screen results, insurance status, and associated diagnosis codes.

A sample of poisoning-related ED visits that were not billed for opioid poisoning from University Hospital 1 was also reviewed for false negatives, including all cases with code 909.*, every other case with code 977.9, every 20th case with code 304.0 or 304.7, and every 10th case with code 305.5, using sequential selection (Table 1).

A standardized reporting form on Excel 2013 was used to abstract data from each visit. Data were entered into Excel 2013 (Microsoft, Redmond, WA, USA) and were analysed with JMP 11 (SAS Institute, Cary, NC, USA) and SAS 9.3 (ibid.).

### Data analysis

Each data abstractor independently assessed whether the primary reason for the visit was an opioid poisoning. Discrepancies were arbitrated by mutual consensus, with a third reviewer (CAS) to resolve any persistent disagreement. Kappa statistics were generated to measure agreement between the first two abstractors.

A multiple regression model was built using data from University Hospital 1 in order to seek a more specific definition of opioid poisoning. The independent variables included in the model were patient age, sex, use of naloxone by EMS or hospital personnel referenced in the triage note, number of ICD-9-CM codes generated, and ordinal position of the opioid-related diagnosis code within the submitted ICD-9-CM code list, and the dependent variable was clinically significant opioid poisoning. The ordinal position of the ICD-9 code within the ICD-9-CM code list was not available using the medical record abstraction system at the other hospitals and they were not included in the multiple regression.

## Results

There were 136 patient visits at University Hospital 1, 63 visits at Community Hospital 1, and 19 visits at Community Hospital 2 that were coded for opioid poisoning (965 series and/or E850 modifiers), of which all charts were reviewed. At University Hospital 2, there were 236 visits with a code for opioid poisoning, of which 141 charts were randomly sampled. Summary statistics of these visits are shown in Table 2. Weighted statistics were calculated, adjusting for the random sampling at University Hospital 2, by multiplying the proportions measured in University Hospital 2 by the total number of visits observed over the number of visits sampled (236/141), so that final statistics reflect the whole population of the two health systems over the study period.

**Table 2:** Demographics and proportion of clinically significant cases among opioid-related ED visits identified by opioid poisoning 965.00, 965.01, 965.02, 965.09 and E850.0-2 ICD-9-CM codes

	University Hospital 1 (*n* = 136)	University Hospital 2 (*n* = 141)	Community Hospital 1 (*n* = 63)	Community Hospital 2 (*n* = 19)	Weighted percentage
Percent male	40 %	51 %	48 %	74 %	48 %
Mean age in years (SD)	43.4 (SD 20.1)	42.1 (SD 17.8)	44.1 (SD 19.2)	31.9 (SD 13.9)	42.3 (SD 10.5)
Percent with chief complaint of poisoning	38 %	27 %	54 %	79 %	36 %
Percent admitted to hospital	70 %	64 %	49 %	26 %	62 %
Percent given naloxone	48 %	50 %	37 %	16 %	46 %
Clinically significant poisoning	70 %	72 %	71 %	74 %	70 %

Overall, 70.1 % (Standard Error 2.4 %) of ED visits receiving a 965 series code were determined to be clinically significant opioid poisoning (the positive predictive value). Standard Error was used to describe the estimate variability because random sampling was employed at University Hospital 2. At University Hospital 1, 18 % of reviewed ED visits that contained a code for poisoning by a non-opioid agent alone were nonetheless determined to be opioid poisonings (Table 3). Among the 136 patients coded for opioid poisoning at University Hospital 1, 3 % had both a history negative for opioid ingestion and a negative toxicologic screening. There was considerable variation between the hospitals in the other characteristics examined including percent of cases with a chief complaint of poisoning, percent of cases in which naloxone was administered, and percent of cases admitted to the hospital. Kappa statistics for inter-rater agreement between data abstractors were 0.58 for University Hospital 1, 0.74 for University Hospital 2, 0.78 for Community Hospital 1 and 0.43 for Community Hospital 2.

**Table 3:** Proportion of ED Visits at University Hospital 1 with non-opioid poisoning ICD-9-CM diagnosis codes that were determined to be opioid-related

Code	909: Late effects of other & unspecified external causes (*n* = 23)	977.9: Poisoning by unspecified drug (*n* = 27)	304.0 or 304.7: Drug dependence (*n* = 11)	305.5: Nondependent opioid abuse (*n* = 17)
Percent male	48 %	37 %	55 %	35 %
Mean age in years (SD)	38.6 (16.9)	27 (16.5)	39.2 (14)	37.2 (12.7)
Percent admitted to hospital	70 %	48 %	73 %	47 %
Percent given naloxone	17 %	7 %	9 %	6 %
Percent with chief complaint of poisoning	17 %	63 %	27 %	41 %
Percent Primary Opioid Poisonings	30 %	22 %	0 %	6 %

In multiple logistic regression, only naloxone use was associated with clinically significant opioid poisoning (*p* < .0001). Overall, 25 % of opioid poisonings determined to be clinically significant received naloxone and 12 % of those not determined to be clinically significant received naloxone.

To assess the range of sensitivity of different parameters, we calculated the positive predictive value of a case definition using the combination of a 965 series diagnosis code and naloxone administration for predicting clinically significant opioid poisoning among cases reviewed at all three hospitals. This case definition increased the positive predictive value to 84 % (*n* = 95 cases with both ICD-9-CM code 965.* and naloxone administration), compared with 46 % positive predictive value with naloxone administration alone (*p* < 0.05). However, the false negative rate for the novel case definition was high (34 %).

## Discussion

Here, we present the first data validating use of ICD-9-CM codes from an ED to detect cases of clinically significant opioid poisoning with reasonable positive predictive value based on medical record review. Given the rapid rise in the number of opioid poisonings, these systems may help to identify locations in which patients are at risk from opioid prescribing or distribution patterns.

The ED serves as an ideal screening point to assess a community for clinically significant overdoses as patients with nonfatal poisoning and concerning symptoms are typically transported for medical evaluation. Our definition of poisoning is easily applied to the ED setting across record systems. Our data show that the majority of those ED visits coded for opioid poisoning were determined to be clinically significant poisoning, and that incorporating free-text searches of triage notes for naloxone administration can improve the positive predictive value of the surveillance.

While few data exist in the literature to establish the positive predictive value of ICD-9-CM codes for clinically significant opioid poisoning, there is substantial variation in ICD-9-CM codes’ ability to identify other injuries, ranging from 64 to 85 % in a recent systematic review [11]. This broad range of accuracies may reflect more or less strict case definitions for particular injuries or poisonings. In the case of opioid poisoning, common coingestions and associated causes of altered mental status (for instance, cancer or chronic painful conditions) may cloud the picture of causality in deaths or adverse events. Prior studies have suggested that patients with comorbid conditions such as cancer may be at lower risk of death from opioid poisoning despite high prescribed doses [12]. The coding uncertainty around polypharmacy poisonings may also contribute to the lowered utility of ICD-9-CM code surveillance for poisonings [13].

The fact that no other factor predicted significant poisoning in our sample reflects the heterogeneity of opioid poisonings with respect to patient initial presentation, demographics, and ultimate disposition, which underscores that opioid poisoning cuts across societal divisions and should be considered in any ED patient with a suggestive history. The heterogeneity of hospital admission rates and naloxone administration by hospital likely points to the differing doses and routes of opioid exposure rather than practice pattern variations, as the hospitals are within closely linked health systems. Distinguishing pure opioid poisoning from other complex causes of respiratory and mental status alteration, including cancer and chronic conditions, may be impossible with the ICD-9-CM coding system.

Most notably, these data are derived from four EDs in North Carolina. These departments may see disproportionately complex cases of opioid poisonings due to referral patterns toward University-affiliated EDs, although there was remarkable consistency among hospitals in our study.

The Kappa statistics for each hospital vary significantly. We hypothesize that the Kappa statistic for University Hospital 2 was higher than that for University Hospital 1 because the reviewers had greater familiarity with the case definition from reviewing University Hospital 1 when they began reviewing University Hospital 2. The low Kappa statistics for Community Hospital 2 can be attributed to that hospital’s unique checkbox-based record system with minimal text (T-System, Dallas, TX, USA) and small sample size.

Since this analysis was conducted on a retrospective cohort of patients based on ICD-9-CM codes, we cannot calculate a true sensitivity and specificity. We attempted to account for this limitation by calculating the positive predictive value of ICD-9-CM for identifying clinically significant cases and by identifying the proportion of opioid poisonings in a selection of cases with ICD-9-CM codes for poisoning by other agents at University Hospital 1 (Table 3). Due to staff resource constraints, these other ICD-9-CM codes were not reviewed at other hospitals.

Our data include only the first 11 ICD-9-CM diagnosis codes generated from a given visit. While it is possible that codes indicating a clinically significant opioid poisoning could appear beyond the 11^th^ diagnosis code position, it is probably not a common occurrence. However, it is likely, particularly with polypharmacy poisonings, that the opioid poisoning code may not be in the first few diagnosis codes listed. To ensure a straightforward case definition, reviewers relied on the clinician’s documented impression in the medical record of whether the case constituted an opioid poisoning. While this approach avoided strict dose-based cut-offs, it allowed for consideration of clinician judgment, which we believe to be more important in real-life situations of uncertainty around both opioid dose and patient response. This algorithm can be flexibly adapted to the more-specific codes contained in the upcoming ICD-10 (which convert approximately to codes T40.1X, T40.2X, T40.3X, T40.60 and T40.69 for opioid poisoning, T50.901S for late effect of poisoning due to drug, medicinal or biological substances, T50.90 for poisoning by other specified drugs and medicinal substances, and F11-F19 for drug abuse and dependence).

## Conclusions

We conclude that, by using the ICD-9-CM codes available in secondary data systems, a real time ED surveillance system can reliably detect clinically significant opioid poisonings across hospital settings. Further work is needed to establish the sensitivity and specificity of the system, which likely varies by geographic context and provider documentation patterns. The results of this study will be used to inform opioid poisoning surveillance efforts using NC DETECT. In future work, real-time ED data will allow for timely investigations and interventions to mitigate harm from geographic and temporal clusters of opioid poisonings.

## Abbreviations

DAWN: Drug Abuse Warning Network
ED: Emergency department
ICD-9-CM: International classification of diseases, 9^th^ edition, clinical modification
NC: North Carolina
NC DETECT: North Carolina Disease Event Tracking and Epidemiologic Collection Tool
NEISS: National Electronic Injury Surveillance System
SEM: Standard Error of the Mean
